# Supplementary material for: Clinical Pharmacists’ Involvement in Pharmacogenomics Testing and Related Services in China
Source: J Pers Med. 2022 Jul 31;12(8):1267. doi: 10.3390/jpm12081267 (PMC9409798; doi:10.3390/jpm12081267)
Supplement: Supplementary file 1 [file jpm-12-01267-s001.zip › jpm-1817052-supplementary.pdf]

Supplementary Materials:

Table S1. Distribution of frequency of PGx tests and department (top ten).

Dept	total	1-10 cases /year		10-30 cases /year		>30 cases /year		Not sure	
		Freq.	%	Freq.	%	Freq.	%	Freq	%
Onco	85	34	40.0	8	9.4	22	25.9	21	24.7
Card	84	29	34.5	11	13.1	24	28.6	20	23.8
Resp	59	25	42.4	10	16.9	7	11.9	17	28.8
ICU	54	25	46.3	2	3.7	11	20.4	16	29.6
Cere/Neur	45	20	44.4	3	6.7	11	24.4	11	24.4
Surg	34	15	44.1	4	11.8	6	17.6	9	26.7
Endo	32	15	46.9	4	12.5	4	12.5	9	28.1
Pedi	32	17	52.1	3	9.4	6	18.8	6	18.8
Dige	30	14	46.7	3	10.0	6	20.0	7	23.3
O&G	27	10	37.0	1	3.7	9	33.3	7	25.9
total	588	241	41.0	61	10.4	129	21.9	157	26.7

Table S2. Self-assessment of the competencies related to PGx testing involved personal practice.

Items		mean	poor (<5)	fair (5-6)	good (7-8)	excellent (9-10)	OR[1] /CI
Evaluate which drugs need PGx testing	Total (n=996)	4.79±2.61	457(45.9)	285(28.6)	170(17.1)	84(8.4)	
	Involved (n=588)	5.27±2.56	218(37.1)	186(31.6)	120(20.4)	64(10.9)	2.34
	No	4.08±2.52	239(58.6)	99(24.3)	50(12.3)	20(4.9)	1.84-2.99
Determine which PGx tests are available at your health care facility	Total (n=996)	5.12±3.09	440(44.2)	219(22.0)	163(16.4)	174(17.5)	
	Involved (n=588)	5.76±3.03	208(35.4)	136(23.1)	107(18.2)	137(23.3)	2.47
	No	4.18±2.95	232(56.9)	83(20.3)	56(13.7)	37(9.1)	1.97-3.14
Interpret the results of PGx testing to the patient/physician	Total (n=996)	5.01±2.80	432(43.4)	243(24.4)	210(21.1)	111(11.1)	
	Involved (n=588)	5.71±2.62	189(32.1)	163(27.7)	150(25.5)	86(14.6)	2.89
	No	4±2.74	243(59.6)	80(19.6)	60(14.7)	25(6.1)	2.27-3.69
Recommend PGx testing to a doctor or patient	Total (n=996)	4.88±2.80	461(46.3)	254(25.5)	159(16.0)	122(12.3)	
	Involved (n=588)	5.45±2.71	224(38.1)	159(27.0)	116(19.7)	89(15.1)	2.26
	No	4.07±2.75	237(58.1)	95(23.3)	43(10.5)	33(8.1)	1.77-2.87
Advise physicians on drug selection, dosage and monitoring	Total (n=996)	5.01±2.74	424(42.6)	273(27.4)	192(19.3)	107(10.7)	
	Involved (n=588)	5.67±2.56	192(32.7)	176(29.9)	135(23.0)	85(14.5)	2.66
	No	4.06±2.71	232(56.9)	97(23.8)	57(14.0)	22(5.4)	2.09-3.39

Annotation：

[1] Respondents who had not engaged in PGx testing and related services were assigned to the non-exposed group, whereas those who had not engaged in PGx testing and related services were assigned to the exposure group.

Table S3. Self-assessment of the competencies related to PGx testing involved the personal practice of frequency of PGx tests (*N*=588).

Items of competency	frequency of PGx tests	poor (<5)	fair (5-6)	good (7-8)	excellent (9-10)	OR[1]	CI
Evaluate which drugs need PGx testing	1-10 cases /year	90(37.3%)	84(34.9%)	53(22.0%)	14(5.8%)		
	10-30 cases /year	25(41.0%)	22(36.1%)	8(13.1%)	6(9.8%)	0.88	0.54-1.46
	>30 cases /year	29(22.5%)	32(24.8%)	36(27.9%)	32(24.8%)	2.80	1.88-4.17
Determine which PGx tests are available at your health care facility	1-10 cases /year	97(40.2%)	55(22.8%)	44(18.3%)	45(18.7%)		
	10-30 cases /year	20(32.8%)	11(18.0%)	15(24.6%)	15(24.6%)	1.49	0.89-2.47
	>30 cases /year	21(16.3%)	30(23.3%)	28(21.7%)	50(38.8%)	2.85	1.92-4.20
Interpret the results of PGx testing to the patient/physician	1-10 cases /year	91(37.8%)	67(27.8%)	55(22.8%)	28(11.6%)		
	10-30 cases /year	14(23.0%)	16(26.2%)	22(36.1%)	9(14.8%)	1.88	1.13-3.10
	>30 cases /year	15(11.6%)	33(25.6%)	43(33.3%)	38(29.5%)	3.57	2.40-5.31
Recommend PGx testing to a doctor or patient	1-10 cases /year	103(42.7%)	57(23.7%)	53(22.0%)	28(11.6%)		
	10-30 cases /year	17(27.9%)	19(31.1%)	19(31.1%)	6(9.8%)	1.5	0.92-2.46
	>30 cases /year	21(16.3%)	40(31.0%)	28(21.7%)	40(31.0%)	2.99	2.02-4.44
Advise physicians on drug selection, dosage and monitoring	1-10 cases /year	90(37.3%)	75(31.1%)	47(19.5%)	29(12.0%)		
	10-30 cases /year	14(23.0%)	20(32.8%)	16(26.2%)	11(18.0%)	1.83	1.10-3.04
	>30 cases /year	16(12.4%)	34(26.4%)	45(34.9%)	34(26.4%)	3.37	2.27-5.00

Annotation:
[1] Respondents who had not engaged in PGx testing at 1-10 cases /year were assigned to the non-exposed group, whereas those who had engaged in PGx testing at 10-30 cases /year or >30 cases /year were assigned to the exposure group.

Area \ Department	Onco	Card	Resp	ICU	Cere/ Neur	Surg	Endo	Pedi	Dige	O&G	Neph	Total
Cardiovascular disease	41	81	48	38	40	26	27	19	26	11	17	433
Rheumatic immune diseases	10	22	24	17	13	11	14	6	7	6	18	172
Infectious diseases	13	9	24	24	6	13	4	9	8	4	10	151
Oncology Therapy	61	11	13	10	6	8	2	4	3	7	2	141
Psychiatry and neurology	9	13	14	13	25	5	4	13	9	5	5	134
Other fields	9	1	8	4	2	3	2	7	2	10	0	61
Pain Treatment	2	5	2	1	1	0	1	0	1	1	0	16
Total	85	84	59	54	45	34	32	32	30	27	22	588

≥50
20-50
10-19
5-9
1-4
0

Figure S1. Distribution of experience in individual PGx tests (multiple choices) and department (top ten) [1].

Annotation:
[1] This is a multiple-choice section.
Individual PGx tests for cardiovascular diseases included those for antihypertensive drugs (ACE I/D polymorphism test; ADRB1 polymorphism test), losartan (CYP2C9*3 polymorphism detection), nitroglycerin (ALDH2*2 polymorphism detection), warfarin (CYP2C9 *3 polymorphism; Vkorc1-1639 G > A polymorphism, CYP4F2*3 polymorphism), coumarin anticoagulants (CYP4F2*3 polymorphism

detection), clopidogrel (CYP2C19*2 and *3 polymorphism detection), simvastatin, cerivastatin (SLCO1B1 521 T>C polymorphism detection), and pravastatin (APOE polymorphism).
Tests for rheumatic immune diseases included those for tacrolimus (CYP3A5*3 polymorphism detection), rasburicase (G6PD gene polymorphism detection), and allopurinol (HLA-B allele test).
Tests for infectious diseases included those for voriconazole (CYP2C19*2 and *3 polymorphism detection), chloroquine (G6PD gene polymorphism detection), dapsone (G6PD gene polymorphism), isoniazid (slow NAT1/NAT2 genotype detection), polymorphisms of peginterferon α-2a, peginterferon α-2b and ribavirin (IFNL3 detection), and abacavir (HLA-B allele test).
Tests for oncology therapies included those for capecitabine (DPYD*2A allele test), tegafur (DPYD*2A allele test), 5-FU (mismatch repair protein deficiency (dMMR) detection, microsatellite instability (MSI) detection, DPYD*2A allele detection), irinotecan (UGT1A1 polymorphism detection), platinum (ERCC1 mRNA expression, TPMT polymorphism detection), anthracycline (TOP2A gene abnormality test), tamoxifen (CYP2D6*10 polymorphism detection), trastuzumab (HER2 gene test), and gemcitabine (RRM1 mRNA expression).
Tests for psychiatric and neurological conditions included those for antipsychotics (ANKK1 RS1800497 polymorphism detection), amitriptyline (CYP2C19*2 and *3 polymorphism detection, detection of CYP2D6*10 polymorphism) and carbamazepine and phenytoin (HLA-B allele detection).
PGx test in other therapeutic fields denotes the detection of other drugs.
The PGx test pertaining to pain treatment is that for celecoxib (CYP2C9*3 polymorphism detection).

Area \ Department	Onco	Card	Resp	ICU	Cere/ Neur	Endo	Surg	Dige	Pedi	Neph	Total
Cardiovascular disease	15	68	29	19	32	19	16	14	12	10	273
Commonly used clinical drug	24	17	16	28	19	8	9	15	15	9	189
Oncology Therapy	60	6	17	9	1	5	5	2	4	1	138
Rheumatic immune diseases	7	13	9	5	1	11	6	4	2	15	95
Psychiatry and neurology	6	4	4	4	18	3	5	4	5	3	77
Infectious diseases	2	2	11	13	0	1	5	3	2	3	57
Endocrine Disease	1	3	0	2	0	9	1	3	2	1	25
Total	83	81	56	50	43	31	31	29	29	22	556

Legend: ≥50; 20-50; 10-19; 5-9; 1-4; 0

Figure S2. Distribution of experience in multiple-gene PGx panel tests (multiple choices) and department (top ten) [1].

Annotation:
[1] This is a multiple-choice section.
Multiple-gene PGx panel tests of cardiovascular diseases include those for common drugs for cardiovascular diseases, anti-arrhythmic drugs, antithrombotic drugs, antihypertensive drugs, and antihyperlipidemic drugs.
The area of commonly used clinical drug includes commonly used clinical drug testing kits and commonly used clinical medicines for children.
The area of oncology therapy includes cancer drug gene testing kit.
The area of rheumatic immune includes anti-gout drug test kit, immunosuppressant test kit and rheumatic disease common medicine test kit.
The area of psychiatry and neurology includes antiepileptic drug test kit, anxiety and depression drug test kit and schizophrenia drug test kit.
The area of infectious diseases includes anti-infective drug test kit.
The area of endocrine disease includes hypoglycemic drug test kit and anti-hyperthyroidism drug test kit.
